# Control of Gray Mold on Clamshell-Packaged ‘Benitaka’ Table Grapes Using Sulphur Dioxide Pads and Perforated Liners

**DOI:** 10.3390/pathogens8040271

**Published:** 2019-11-28

**Authors:** Osmar Jose Chaves Junior, Khamis Youssef, Renata Koyama, Saeed Ahmed, Allan Ricardo Dominguez, Débora Thaís Mühlbeier, Sergio Ruffo Roberto

**Affiliations:** 1 Agricultural Research Center, Londrina State University, Londrina 86057-970, Brazil; osmarjcj@gmail.com (O.J.C.J.); emykoyama@hotmail.com (R.K.); saeeddikhan@gmail.com (S.A.); allandomingez@hotmail.com (A.R.D.); muhlbeierdebora@gmail.com (D.T.M.); 2 Agricultural Research Center, Plant Pathology Research Institute, 9 Gamaa St., Giza 12619, Egypt

**Keywords:** Brazil, gray mold, packaging technology, postharvest treatments, table grapes

## Abstract

The use of vented clamshells has become popular in the packaging of grapes for local and international markets. The aim of this study is to evaluate the postharvest preservation of ‘Benitaka’ table grapes individually packaged in vented clamshells using different types of SO_2_-generating pads and perforated plastic liners during cold storage. A completely randomized design with four replications in a two-factor arrangement with an additional treatment [(4 × 3) + 1] was used. The trials were carried out under two situations: Artificial or natural infections with *Botrytis cinerea*, which is the causal agent of gray mold on table grapes. The incidence of gray mold, shattered berries, and stem browning were evaluated at 30 and 45 days of cold storage at 1 ± 1 °C and 3 days of shelf-life at 22 ± 1 °C after the period of cold storage. Mass loss and berry firmness were also examined at the end of the cold storage period. The use of dual-release SO_2_-generating pads containing 5 or 8 g of a.i. and slow-release pads with 7 g of a.i. was effective in controlling the incidence of gray mold in grapes packaged in vented clamshells and kept under cold storage for up to 45 days. Under these storage conditions, perforated plastic liners with 0.3% ventilation area or micro-perforated liners with 1.0% ventilation area reduced the percentage of mass loss and shattered berries.

## 1. Introduction

One of the most important varieties of table grapes cultivated in tropical areas is the Benitaka (*Vitis vinifera* L.), a cultivar that originated from a somatic mutation of the ‘Italia’ table grape and has attracted the attention of many growers in the last few years [1,2]. Under natural conditions, the color index of ‘Benitaka’ table grape grows in subtropical areas ranging 3.1–4.6, which means that they red-violet skin in color [3]. Table grapes with good productive performance and characteristics of consumption, such as healthiness and freshness, few shattered berries, green rachis, and intense berry color, are preferred. 

The grape productive chain is very demanding in terms of preserving fruit quality. Additionally, maintaining the characteristics of harvested grapes and enabling an increase in shelf-life is important as the fruit is subjected to long storage periods before reaching its final destination, and there are risks of various postharvest losses [4]. Grapes are exposed to different conditions between harvesting and consumption. During this period, several factors can affect fruit quality, including handling damage, loss of water, and pathogen attack [5]. These factors can cause a loss of quality and can interrupt the sale of the grapes, since local and export markets require a high standard of fruit quality. Aiming to maintain the postharvest quality of fresh grapes, the packaging of bunches in vented clamshells has become an innovative alternative that meets these needs in both local and export markets. This form of packaging prevents physical contact of the bunch with the external environment, and the fruit has less damage and prolonged shelf-lives, since the consumer does not handle the grapes directly [6]. Besides preventing manipulation, vented clamshells are of the utmost convenience for the consumer, they can be easily accommodated, and enable greater integrity of the grape bunch until its final destination is reached [7,8]. The use of clamshells provides a success combination of practicality, protection, and marketing, leading to increases in sales in several markets [9]. Nevertheless, more attention should be given to the used plastic materials, which are designed for fruit packing.

However, *Botrytis cinerea*, the causal agent of gray mold, causes significant postharvest loss of grapes, even when bunches are packaged in clamshells. The control of this disease is very difficult since postharvest treatments with synthetic fungicides are not allowed in several countries [10]. Generally, in Parana state (Brazil), there are seven active ingredients to be used against postharvest grape diseases, including captan, chlorothalonil, iprodione, mancozeb, pyrimethanil, procymidone, and thiophanate-methyl [1]. In the main table grape growing areas of this state, synthetic fungicide based on iprodione at 0.2% (applying three times during the season) is the main method to control the disease before harvest. Many of the generally recognized as safe compounds (GRAS), including organic and inorganic salts, are allowed for the Brazilian and external markets. In the European market there are two commercial products based on the salts that are available for controlling postharvest diseases of fruit, namely Bioprotege (based on sodium bicarbonate and silicium dioxide) and Karma (based on potassium bicarbonate). In fact, several ecofriendly and safe alternatives were used to control the gray mold of grapes, including biological control agents and natural salts to obtain high-quality grapes and wines with elevated standards of food security [11,12,13].

One of the most widely used postharvest techniques to prevent the development of this disease is cold storage, which is effective for increasing fruit longevity, and this technique, combined with other packaging and storage practices, enables a longer grape shelf-life before consumption [14]. The use of SO_2_-generating pads inside the carton boxes has demonstrated good performance for controlling postharvest diseases during cold storage [10,15]. The pads are composed of sheets containing sodium or potassium metabisulfite, and SO_2_ gas is generated by the reaction of these compounds with the humidity present in the air. Different types of SO_2_-generating pads provide different release amounts of SO_2_, such as dual and slow-release pads [16].

The selection of SO_2_-generating pads should be judicious to maintain the quality of the harvested product during transport to its final destination. Additionally, the level of active ingredients must be appropriate so as not to damage the fruit or impair its flavor [17]. The main import markets of fresh grapes, such as the European Union and the United States, established tolerance levels for the use of SO_2_ in postharvest management because high concentrations of this gas can be harmful to humans and the environment [18,19].

To further improve the efficiency of SO_2_-generating pads in the cold storage of table grapes, it is necessary to use these pads in combination with perforated plastic liners to facilitate their circulation in the packaging and also avoid loss of fruit mass. These films are composed of low-density polyethylene made from single or liner sheets [20,21]. The perforated plastic liners must be permeable with perforations that allow an adequate ventilation area that varies according to its level of permeability [22,23].

According to the available literature, little is known about the interaction between different types of SO_2_-generating pads and perforated liners in the postharvest cold preservation of ‘Benitaka’ table grapes, mainly regarding the incidence of gray mold when grapes are individually packaged in vented clamshells. The objective of this study was to evaluate the combinations of different types of SO_2_-generating pads and perforated plastic liners on gray mold incidence, shattered berries, stem browning, mass loss, and firmness of ‘Benitaka’ table grapes packaged in clamshells.

## 2. Materials and Methods

### 2.1. Location of the Experiments and Materials used

The bunches of ‘Benitaka’ table grape were obtained from a commercial property located in Cambira, PR, Brazil (23°35′ S, 51°34′ W, elevation 1017 m), in a vineyard that has history of the occurrence of gray mold (*Botrytis cinerea*), downy mildew (*Plasmopara viticola*), powdery mildew (*Erysiphe necator*), anthracnose (*Elsinoe ampelina*), and ripe rot (*Colletotrichum gloeosporioides*). The region is classified as subtropical (Cfa), according to Köppen, with an average annual temperature of 20.7 °C and annual rainfall of 1600 mm [24]. The vines were 10 years old and were grafted on the rootstock ‘IAC 766 Campinas’ trained on an overhead trellis system protected by a black screen with 18% shading. The harvest was performed during the 2018 season when the total soluble solid content of the grapes reached 14 °Brix.

The study was conducted in a two-factor arrangement with an additional treatment [(4 × 3) + 1], with four replicates of each of the treatments, as follows: First, sulphur dioxide (SO_2_) generating pads of slow release containing 4 and 7 g of the active ingredient (a.i.) each, and dual release containing 5 and 8 g of the i.a. each. Second, perforated liners with varying ventilation areas at 0.3%, 0.9%, and 1% area. Third, an additional treatment with a standard microperforated plastic liner (1%) and without sulphur dioxide pads. Each replicate had 10 clamshells totalling 40 clamshells per treatment.

The SO_2_-generating pads (Uvas Quality Grape Guard^®^, Suragra S.A., San Bernardo, Chile) were 26 × 46 cm in size and incorporated 98% of the a.i., sodium metabisulfite (Na_2_S_2_O_5_). The slow-release pad containing 7 g of the a.i. was made with two polymer films containing SO_2_ in a solvent-free wax matrix, allowing contact with the grapes. The slow-release pad containing 4 g of the a.i. was made with coextruded polymer film, with a low risk of bleaching the fruit due to the low diffusion of SO_2_. The dual-release pad containing 5 g of the a.i. was made with extruded polymer film and 100% virgin paper pulp obtained by a mechanical process, with doses of fast and slow phases of 1 and 4 g of the a.i., respectively. The dual-release pad containing 8 g of a.i. was made from coextruded paper with polyethylene and 100% virgin paper pulp obtained by mechanical means, with doses of fast and slow phases of 1 and 7 g of the a.i., respectively. 

The different perforated plastic liners (Suragra S.A., San Bernardo, Chile) were prepared with high-density plastic and master batch, with dimensions of 95 × 65 cm and a thickness of 12 µm. The macroperforated liners (0.3% and 0.9% ventilation area, respectively) had vent holes with dimensions of 70 × 90 mm each, whereas the plastic liner with 1.0% ventilation area was microperforated. 

Bunches were selected free of any disorders or any decayed berries and trials were carried out under two situations: Artificially and naturally occurring infections.

### 2.2. Artificial and Natural Occurring Infections

#### 2.2.1. Inoculation of Grapes with *B. cinerea*

**Figure 1:**
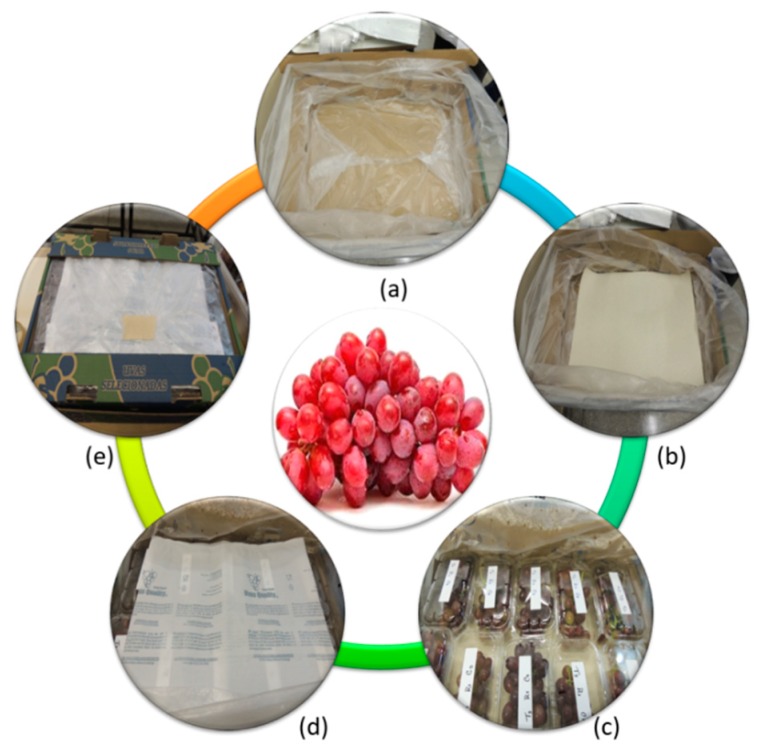
Steps in preservation and packaging of grapes using SO_2_-generating pads and perforated plastic liners. a: Accommodation of plastic liner inside a corrugated box; b: Placement of absorbent paper sheet; c: Arrangement of grapes clamshells in the box; d: Accommodation of the SO_2_-generating pads over the clamshells; e: Closure and seal of the perforated plastic liner ready for cold storage.

Fungal suspension was prepared according to the standard protocol using a *B. cinerea* isolate (BCUEL-1) taken from infected grapes with typical symptoms of the disease, purified, and identified morphologically and molecularly, according to Youssef and Roberto [25]. Fungal inoculum concentration was normalized to 10^6^ conidia mL^−1^ using haemecytometer (Neubauer Boeco, Hamburg Germany). Grape bunches were pooled together, surface sterilized by immersion in sodium hypochlorite solution (1%) for 2 min, rinsed with sterile distilled water and air-dried. After drying, bunches were inoculated by being sprayed with the suspension of fungal conidia. Approximately 450 mL of inoculum was sprayed on about 100 kg of grapes with a compressed plastic sprayer. After drying, the bunches were standardized and were accommodated in 20 × 10 cm vented clamshells (10 holes distribution) with 0.5 kg capacity, which were wrapped in perforated plastic liners that had different perforation sizes and ventilation areas according to the treatment description. Grapes wrapped in clamshells were placed inside a corrugated carton boxes measuring 100 × 60 × 40 cm, with a storage capacity of 10 clamshells each. Four replicates were used per treatment. On the bottom, a unilaminar sheet of moisture-absorbing paper, measuring 33 × 46 cm with a density of 50 g m^−2^, was placed. In each box, one SO_2_-generating pad was placed above the clamshells. All packing steps are shown in Figure 1.

#### 2.2.2. Natural Infection

The second part of the grape bunches were standardized and accommodated in clamshells with a 0.5 kg capacity, as described above, and were subjected to the same treatments without any fungal inoculation. For both artificial and natural infections, the statistical design employed was a completely randomized design with four replicates, with each plot consisting of 10 clamshells arranged in a corrugated cardboard box. Thereafter, the corrugated boxes were placed in a cold chamber at 1 ± 1 °C for 45 days, followed by 3 days of shelf-life at 22 ± 1 °C with relative humidity above 90%.

### 2.3. Postharvest Quality Assessments

The treatments were evaluated after 30 and 45 days of cold storage with the following variables being assessed: Gray mold incidence (%), shattered berries, stem browning, and mass loss. For artificial trial, only the incidence of gray mold was assessed. The incidence of gray mold was calculated by the following formula: Incidence (%) = (number of affected berries/total of berries) × 100 [25].

The percentage of broken/shattered berries was evaluated by counting the loose berries from the bunch inside the clamshells, and was expressed as a percentage. Stem browning was evaluated through visual assessment according to the methodology described by Ngcobo et al. [17], assigning notes in accordance with the level of darkness: (1) Fresh and green, (2) some light browning, (3) significant browning, and (4) severe browning. 

The bunch mass loss was obtained by weighing the bunches at the initial time of storage and at the time of each evaluation, in accordance with Youssef and Roberto [25]: Mass loss (%) = [(initial mass − mass at examined date)/initial mass] × 100.

The berry firmness (N) was only evaluated after 45 days of cold chamber and was measured with the texture analyzer TA.XT*plus* (Stable Micro Systems, Surrey, England) at the equatorial position of 10 berry samples from each replicate (totalizing 40 berries per each treatment). Each berry was placed on the base of the analyzer and compressed using a cylindrical probe (35 mm diameter, P35). A constant force of 0.05 N at a speed of 1.0 mm s^−1^ was then used to deform the berry by 20%, and the result was expressed as firmness loss in relation to the initial firmness [26].

After the 45 days of cold storage, the boxes were further stored at 22 ± 1 °C for 3 days and gray mold incidence, shattered berries, and stem browning were assessed.

### 2.4. Statistical Analysis

Data were subjected to analysis of variance, and the means were compared by Tukey’s HSD at 5% of the level of significance in *R* software. To evaluate the relationship between the treatments and postharvest attributes, means were further subjected to Principal Component Analysis (PCA) in *FactorMineR*.

## 3. Results and Discussion

### 3.1. Artificial Inoculation with B. cinerea

The percentage of gray mold incidence in ‘Benitaka’ table grapes artificially inoculated with *B. cinerea* is shown in Table 1. Overall, there was no interaction between the different types of SO_2_-generating pads and perforated plastic liners; however, a significant reduction of gray mold incidence was observed when grapes were stored with slow release (7 g) and dual release 5 and 8 g SO_2_-generating pads. Those treatments kept their performance for the three evaluating intervals (30 and 45 days of cold storage and 3 days of shelf-life after the period of cold storage). No significant difference was detected among the different perforated plastic liners. In contrast, the highest percentage of gray mold incidence (22.9%, 71.1%, and 98.6% after 30, 45 days in cold chamber, and after 3 days at shelf-life, respectively) was observed when a slow-release pad containing 4 g of a.i. was used (Table 1).

The slow release associated with the low quantity of gas in this treatment may have compromised the initial control of the fungus inoculum, and fungal growth was controlled more efficiently when a slow-release pad containing 7 g of a.i. was employed or any of the dual-release pads (5 or 8 g of a.i.). This result can be explained by the different types of coatings, release forms, and concentrations of a.i. in the pads [27]. In addition, even under artificial inoculation of *B. cinerea* on grapes, the clamshells did not seem to act as a barrier for an even circulation of the SO_2_ generated by the pads in the boxes, what can be confirmed by the high efficient control of the disease observed, especially when dual-release pads were used, irrespective of the different liner ventilation areas. Additionally, dual release pads might offer critical protection against decay when natural decay is medium or high.

### 3.2. Natural Occurring Infection of Gray Mold

No significant interactions were observed between the different SO_2_-generating pads and the perforated plastic liners for all variables assessed in ‘Benitaka’ grapes with naturally occurring infection of *B*. *cinerea*. The natural gray mold incidence of ‘Benitaka’ table grapes is shown in Table 2. The dual release pad containing 5 or 8 g of a.i. significantly reduced the percentage of mold incidence after 30 and 45 days of cold storage and also after 3 days of shelf-life. In particular, the dual release with 8 g of a.i. completely suppressed the development of gray mold at all evaluating periods. A low incidence of gray mold was observed for grapes stored with slow release containing 7 g of a.i. (Table 2). These differences can be attributed to different ways of gas release, concentrations of a.i., and materials of the SO_2_-generating pads, which provide different permeability [28] and control levels. The slow SO_2_-releasing pad (7 g) is designed with two films of polymers that contain the SO_2_ in an array of solvent-free wax, and for this reason, depending of the grape cultivar sensitivity, may contact the bunches of grapes without increasing the incidence of bleaching of the berries, whereas 4 g of a.i. is designed with coextruded polymer film. Thus, the greater efficiency of the slow SO_2_-releasing pad (7 vs. 4 g) can be explained by the higher concentration of a.i. and the different coating materials of the pads.

The obtained performance of dual-release pads against gray mold was possibly due to contact with air moisture, which resulted in the release of a high amount of gas in the first 48 h of storage (1 g of a.i.), thus eliminating any fungal conidia. After this period, the emission of the gas became slow and steady. However, there were no significant differences between the two dual-release SO_2_-generating pads tested. After 45 days of cold storage, a low incidence of gray mold in the grapes was observed when the dual-release pads containing 5 g of a.i. were used, which did not occur when the 8 g dual-release pads were used (Table 2). In this case, the highest dose of SO_2_ could keep ‘Benitaka’ grapes completely free of disease development during the storage period.

On the other hand, despite emitting a constant amount of gas from the beginning to the end of the storage period, the slow release pad containing 7 g of a.i. did not lead to a complete absence of gray mold in the grapes, which can also be influenced by the natural occurrence of the fungus and the types of packaging employed [29,30,31]. The different plastic liners evaluated had no influence on this feature in the periods evaluated, however, it was found that the bunches submitted to a combination of factors displayed a lower mean incidence of gray mold compared to those in the additional treatment, which confirmed the need for SO_2_-generating pads in the control of gray mold of ‘Benitaka’ table grapes during cold storage [4].

After three days of shelf-life, differences in the incidence of gray mold were observed between the different types of SO_2_-generating pads (Table 2). During this period, the incidence of the disease remained low, which can be explained by the retention and distribution of the gas released by the SO_2_-generating pads [21]. The highest incidence were observed when the grapes were treated with slow SO_2_-generating pads, with the highest mean observed when 4 g of a.i. was used, which indicates that the amount of gas released was insufficient and did not impede the development of the fungus. In contrast, the dual-release pads resulted in a more effective control of gray mold, which can be attributed to their fumigant action in the first 48 h of storage [32].

Generally, the low natural rot observed in this study was probably due to the field fungicide program used. As a standard chemical control, farmers in this state frequently apply iprodione at 0.2% three times during the season (after flowering, at pre-bunch closure, at veraison). Since the experiments were carried out on naturally occurring infections, rot incidence in the control treatment was not very high. With naturally occurring infection, it is possible to verify the efficacy of the control methods on every type of infection, namely latent, quiescent, and incipient infections, not only on wound infections. Additionally, evaluating the efficacy of candidate control methods on natural infections is necessary and essential when researchers are looking for a commercial application [33,34,35,36].

### 3.3. Grape Quality

Overall, there was no significant interaction between the different SO_2_-generating pads and plastic liners for all grape quality parameters. Shattered berries and stem browning were evaluated 30, 45 days of cold storage, and also 3 days of shelf-life after the period of cold storage, and the results are shown in Table 3 and Table 4. There was no effect of the different SO_2_-generating pads on shattered berries, however, the highest mean was observed when the plastic liner with 0.9% ventilation area was used, followed by those with a 1.0% and 0.3% ventilation area (Table 3). This is possibly due to the relationship between the ventilation area and a potential reduction in humidity inside the boxes, leading to greater dehydration, mass loss, and concomitantly more shattered berries [37].

There were no significant differences between the different SO_2_-generating pads regarding the shattered berries and stem browning at 3 days of shelf-life after the period of cold storage (Table 3 and Table 4). The low concentration of SO_2_ treatment was more effective throughout storage in regards to the incidence of fungal decay and stem browning [28]. However, significant differences were found among the perforated plastic liners. In particular, a 0.9% ventilation area resulted in higher means for these evaluated characteristics (1.18% and 2.40% for shattered berries and stem browning, respectively). The obtained results herein can be explained by the greater mass loss of the grape bunches provided by this treatment [37]. It was also verified that the means of the combination of the factors were superior to that of the additional treatment for these two variables.

The percentage of mass loss was determined after 30 and 45 days of cold storage, while firmness was only evaluated after 45 days of cold storage (Table 5). Overall, no significant difference was observed among treatments with different SO_2_-generating pads regarding the mass loss and firmness of the grapes. However, the highest mean of mass loss in perforated plastic liners occurred when a 0.9% ventilation area was used, and this difference was more pronounced after 45 days of cold storage (1.81%). The increase in ventilation area of the plastic liner can result in the reduction of moisture in the packaging and greater dispersion of SO_2_, which explains the higher dehydration of bunches, and consequently a greater mass loss. Even though the ventilation area of the 0.9% and 1.0% liners was similar, the type of perforation of each one of them should be considered (macro and micro-perforated, respectively).

Market chains used to pile mounds of grapes on a shelf and shoppers picked a bunch or two off the top, but under these circumstances, losses were higher, since grapes last longer when air circulates around them to prevent gray mold. The use of vented clamshells to pack and store grapes has become a new trend, not only for exported grapes, but also for domestic markets resulting in a more efficient way to provide fresh fruits of superior quality to consumers. Packing ‘Benitaka’ grapes in vented clamshells is a novelty, since this cultivar is usually packaged in plastic bags or even piled in carton boxes. According to our findings, the ‘Benitaka’ bunches did not seem to have any negative influence, in terms of the efficiency of the SO_2_-generating pads combined with perforated plastic liners, to maintain the quality of fresh grapes for an extended period during cold storage. When closed, the lids of clamshell containers did not impede SO_2_ diffusion into the grapes since some types of pads, especially the dual release, were able to provide an excellent control of gray mold. Regarding the color index, no negative effects of the different types of SO_2_-generating pads and perforated plastic liners were observed.

Using Principal Component Analysis (PCA), it was possible to confirm these findings and to assess the treatments regarding their performance in the postharvest preservation of ‘Benitaka’ table grape with a higher proximity.

### 3.4. Principal Component Analysis (PCA)

**Figure 2:**
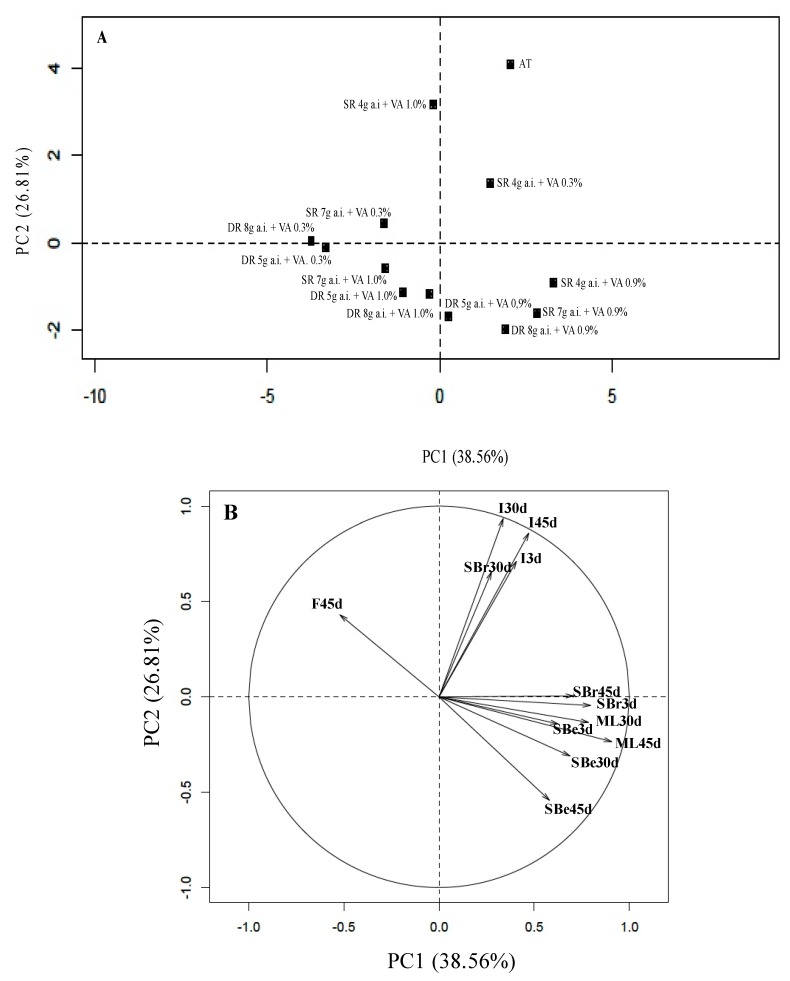
Principal Component Analysis (PCA) of gray mold incidence and postharvest attributes of ‘Benitaka’ table grapes after 30 and 45 days of cold storage, and after 3 days of shelf-life, individually packaged in clamshells with different SO_2_-generating pads and perforated plastic liners. (**A**): treatments dispersion according to the scores of the principal components. (**B**): variables arrangement according to the scores of the principal components. SR: slow release of SO_2_; DR: dual release of SO_2_; a.i.: active ingredient; VA: ventilation area; AT: additional treatment; I: incidence of gray mold; SBe: shattered berries; SBr: stem browning; ML: mass loss; F: firmness; d: days.

Grapes treated with dual-release SO_2_-generating pads clustered opposite to the additional treatment, which was expected because this consisted only of cold storage of grapes packaged in plastic liners with a 1% ventilation area, without SO_2_-generating pads to control gray mold development (Figure 2A). Nevertheless, the good performance of the slow-release pad with 7 g of a.i. stands out, and this treatment was grouped similarly to those with the dual-release SO_2_-generating pads. Thus, it is evident that the use of appropriate postharvest packaging and preservation techniques allows for the maintenance of the main properties of table grapes under suitable conditions for prolonged periods [16]. On the other hand, a similar performance was observed after the additional treatment with the slow SO_2_-generating pads containing 4 g of a.i., which can be explained by the fact that the amount of gas released in this treatment was insufficient to control gray mold development. Furthermore, the slow-release property of this type of pad, which only releases a constant amount of gas throughout the storage period, unlike the dual-release pads that also contains a fast release phase in the first 48 h [20], resulted in the loss of quality of ‘Benitaka’ grapes. The type of perforated plastic liners allowed the clustering of treatments, revealing two distinct groups: The first one was composed of those in which SO_2_-generating pads were used in combination with the plastic liner with a 0.9% ventilation area; and the second one in which the different generating pads were used in combination with the plastic liner with 1.0% ventilation area. This grouping highlighted the distinction between types of perforations of plastic liners [37] because both groups had similar ventilation areas. Due to its microperforation, the plastic liner with a 1.0% ventilation area possibly retained the gas more efficiently than the 0.9% ventilation area (macroperforated) liner, which maked its effectiveness similar to the plastic liner with a 0.3% ventilation area [16].

Only the treatments consisting of slow SO_2_-generating pads with 4 g of a.i. combined with liners with 0.3 or 1.0% ventilation area were distinguished from others with the same ventilation area, with similar results to the additional treatment. This observation allows us to infer that these treatments tend to resemble the additional treatment, and the type of SO_2_-generating pad possibly responsible for this similarity. However, all types of pads combined with the plastic liner with 0.9% ventilation area had similar results to the additional treatment. The natural incidence of gray mold presents different behavioral characteristics in ‘Benitaka’ table grapes. Moreover, these characteristics are associated with the grouped treatments (Figure 2B). Other characteristics, such as shattered berries, stem browning, and mass loss were correlated with the treatment with a perforated plastic liner (0.9% ventilation area), whereas the firmness of berries revealed an opposite effect, correlating more to the treatments in which the dual-release pads were employed.

## 4. Conclusions

The objective of the current research was to assess the postharvest preservation of ‘Benitaka’ table grapes packaged in vented clamshells using different types of SO_2_-generating pads and perforated plastic liners under cold storage. Stimulating the current commercial situation, the use of dual-release SO_2_-generating pads containing 5 or 8 g of a.i., as well as the slow-release of 7 g a.i., is effective to control the development of gray mold on ‘Benitaka’ table grapes packaged in vented clamshells and kept in a cold chamber at 1 ± 1 °C for up to 45 days. In these storage conditions, the use of plastic liners with 0.3% ventilation area or the microperforated liner with 1.0% ventilation area resulted in a decreased mass loss of grape bunches and shattered berries. After the cold storage period, when grapes were kept at 22 ± 1 °C for 3 days, these SO_2_-generating pads also resulted in a lower incidence of gray mold, shattered berries, and stem browning. Finally, no negative effect was observed in terms of fruit quality. The obtained results may clarify the interaction between different types of SO_2_-generating pads and perforated liners in the postharvest cold preservation of ‘Benitaka’ table grape.

## Figures and Tables

**Table 1:** Incidence of gray mold (% of affected berries) in ‘Benitaka’ table grapes after 30 and 45 days of storage in cold chamber at 1 ± 1 °C and 3 days at shelf-life at 22 ± 1 °C after the period of cold storage, individually packaged in clamshells with different SO_2_-generating pads and perforated plastic liners, inoculated with 10^6^ spores mL^−1^ *Botrytis cinerea*.

Type of SO_2_ Release and Amount of SO_2_ in Pads (A)	Incidence of Gray Mold (% of Affected Berries)		
	After 30 Days in Cold Chamber	After 45 Days in Cold Chamber	After 3 Days at Shelf-Life
Slow release—4 g	22.86 ± 7.05 a	71.09 ± 9.51 a	98.60 ± 9.64 a
Slow release—7 g	4.68 ± 2.15 b	19.67 ± 6.08 b	25.80 ± 9.52 b
Dual release—5 g	0.00 ± 0.00 b	1.02 ± 1.02 b	2.23 ± 1.51 b
Dual release—8 g	0.00 ± 0.00 b	0.00 ± 0.00 b	0.00 ± 0.00 b
**Ventilation area of perforated plastic liners (B)**			
0.3%	11.17 ± 5.99 a	25.11 ± 9.87a	34.74 ± 11.70 a
0.9%	4.58 ± 3.60 a	22.39 ± 7.92a	32.89 ± 11.53 a
1.0%	4.90 ± 3.49 a	21.33 ± 9.13a	34.94 ± 10.40 a
**Contrast of the additional treatment with the factorial**			
Additional treatment	13.16 ± 9.47 a	50.85 ± 24.13 a	86.19 ± 18.94 a
Factorial	6.88 ± 4.50 a	22.95 ± 8.86 a	34.19 ± 11.04 b
F (A)	7.74 *	10.48 *	13.01 *
F (B)	1.20 ^ns^	0.05 ^ns^	0.00 ^ns^
F (A × B)	0.66 ^ns^	0.46 ^ns^	0.62 ^ns^
F (additional treat. × factorial)	0.79 ^ns^	2.25 ^ns^	4.15 *
CV %	30.34	5.64	5.24

Means within columns followed by the same letters are not statistically different, as determined by Tukey’s test (* *p* ≤ 0.05). ns: not significant. CV: coefficient of variation. Original data were transformed by $\sqrt{x+0.5}$.

**Table 2:** Natural incidence of gray mold (% of affected berries) of ‘Benitaka’ table grapes after 30 and 45 days of storage in cold chamber at 1 ± 1 °C and 3 days at shelf-life at 22 ± 1 °C after the period of cold storage, individually packaged in clamshells with different SO_2_-generating pads and perforated plastic liners.

Type of SO_2_ Release and Amount of SO_2_ in Pads (A)	Incidence of Gray Mold (% of Affected Berries)		
	After 30 Days in Cold Chamber	After 45 Days in Cold Chamber	After 3 Days at Shelf-Life
Slow release—4 g	0.31 ± 0.11 a	0.92 ± 0.20 a	2.68 ± 0.42 a
Slow release—7 g	0.03 ± 0.04 b	0.35 ± 0.12 b	0.35 ± 0.07 b
Dual release—5 g	0.00 ± 0.00 b	0.04 ± 0.00 b	0.04 ± 0.00 b
Dual release—8 g	0.00 ± 0.00 b	0.00 ± 0.04 b	0.00 ± 0.00 b
**Ventilation area of perforated plastic liners (B)**			
0.3%	0.10 ± 0.07 a	0.37 ± 0.17 a	0.69 ± 0.34 a
0.9%	0.03 ± 0.04 a	0.26 ± 0.11 a	0.66 ± 0.28 a
1.0%	0.12 ± 0.08 a	0.35 ± 0.18 a	0.95 ± 0.53 a
**Contrast of the additional treatment with the factorial**			
Additional treatment	0.59 ± 0.23 a	1.45 ± 0.57 a	1.46 ± 0.55 a
Factorial	0.08 ± 0.07 b	0.32 ± 0.16 b	0.71 ± 0.39 a
F (A)	5.41 *	11.19 *	55.32 *
F (B)	0.65 ^ns^	0.16 ^ns^	0.43 ^ns^
F (A × B)	0.66 ^ns^	0.85 ^ns^	0.82 ^ns^
F (additional treat. × factorial)	17.43 *	18.96 *	2.58 ^ns^
CV %	19.74	32.44	47.69

Means within columns followed by the same letters are not statistically different as determined by Tukey’s test (* *p* ≤ 0.05). ns: not significant. CV: coefficient of variation. Original data were transformed by $\sqrt{x+0.5}$.

**Table 3:** Shattered berries (%) of ‘Benitaka’ table grapes after 30 and 45 days storage in a cold chamber at 1 ± 1 °C and 3 days at shelf-life at 22 ± 1 °C after the period of cold storage, individually packaged in clamshells with different SO_2_-generating pads and perforated plastic liners.

Type of SO_2_ Release and Amount of SO_2_ in Pads (A)	Shattered Berries (%)		
	After 30 Days in Cold Chamber	After 45 Days in Cold Chamber	After 3 Days at Shelf-Life
Slow release—4 g	0.22 ± 0.09 a	0.65 ± 0.23 a	1.11 ± 0.06 a
Slow release—7 g	0.34 ± 0.06 a	0.50 ± 0.12 a	1.05 ± 0.05 a
Dual release—5 g	0.28 ± 0.12 a	0.55 ± 0.20 a	1.06 ± 0.04 a
Dual release—8 g	0.20 ± 0.10 a	0.43 ± 0.11 a	1.17 ± 0.05 a
**Ventilation area of perforated plastic liners (B)**			
0.3%	0.15 ± 0.09 a	0.34 ± 0.20 b	1.03 ± 0.03 b
0.9%	0.36 ± 0.09 a	0.81 ± 0.14 a	1.18 ± 0.06 a
1.0%	0.27 ± 0.09 a	0.45 ± 0.13 ab	1.08 ± 0.06 ab
**Contrast of the additional treatment with the factorial**			
Additional treatment	0.38 ± 0.38 a	0.11 ± 0.12 a	1.29 ± 0.05 a
Factorial	0.26 ± 0.09 a	0.53 ± 0.17 a	1.10 ± 0.05 b
F (A)	0.43 ^ns^	1.04 ^ns^	1.36 ^ns^
F (B)	1.53 ^ns^	3.61 *	3.81 *
F (A × B)	0.30 ^ns^	0.18 ^ns^	1.40 ^ns^
F (additional treat. × factorial)	0.07 ^ns^	2.23 ^ns^	5.23 *
CV %	21.86	2.76	15.85

Means within columns followed by the same letters are not statistically different as determined by Tukey’s test (* *p* ≤ 0.05). ns: not significant. CV: coefficient of variation. The original data were transformed by $\sqrt{x+0.5}$.

**Table 4:** Stem browning scores of ‘Benitaka’ table grapes after 30 and 45 days of storage in a cold chamber at 1 ± 1 °C and 3 days at shelf-life at 22 ± 1 °C after the period of cold storage, individually packaged in clamshells with different SO_2_-generating pads and perforated plastic liners.

Type of SO_2_ Release and Amount of SO_2_ in Pads (A)	Stem Browning ^a^		
	After 30 Days in a Cold Chamber	After 45 Days in a Cold Chamber	After 3 Days at Shelf-Life
Slow release—7 g	1.00 ± 0.00 a	1.28 ± 0.11 ab	2.08 ± 0.09 a
Slow release—4 g	1.00 ± 0.00 a	1.30 ± 0.06 a	2.33 ± 0.07 a
Dual release—5 g	1.00 ± 0.00 a	1.11 ± 0.07 ab	2.06 ± 0.08 a
Dual release—8 g	1.05 ± 0.00 a	1.05 ± 0.04 b	2.30 ± 0.08 a
**Ventilation area of perforated plastic liners (B)**			
0.3%	1.00 ± 0.00 a	1.31 ± 0.07 a	2.10 ± 0.05 b
0.9%	1.00 ± 0.00 a	1.12 ± 0.09 a	2.40 ± 0.09 a
1.0%	1.00 ± 0.00 a	1.12 ± 0.07 a	2.08 ± 0.08 b
**Contrast of the additional treatment with the factorial**			
Additional treatment	1.05 ± 0.05 a	1.20 ± 0.08 a	2.19 ± 0.13 a
Factorial	1.00 ± 0.00 a	1.18 ± 0.08 a	2.07 ± 0.08 b
F (A)	0.14 ^ns^	3.51 *	4.29 *
F (B)	1.08 ^ns^	0.80 ^ns^	8.46 *
F (A × B)	0.07 ^ns^	1.59 ^ns^	0.55 ^ns^
F (additional treat. × factorial)	12.00 ^ns^	0.01 ^ns^	18.39 *
CV %	27.41	21.95	13.14

Means within columns followed by the same letters are not statistically different as determined by Tukey’s test (* *p* ≤ 0.05). ns: not significant. CV: coefficient of variation. ^a^ Stem browning scores: (1) fresh and green, (2) some light browning, (3) significant browning, and (4) severe browning [14].

**Table 5:** Mass loss (%) and berry firmness (N) of ‘Benitaka’ table grapes individually packaged in clamshells with different SO_2_-generating pads and perforated plastic liners. Mass loss was evaluated after 30 and 45 days of storage in a cold chamber, while firmness was evaluated after 45 days of storage in a cold chamber at 1 ± 1 °C.

Type of SO_2_ Release and Amount of SO_2_ in Pads (A)	Mass Loss (%)		Firmness (N)
	After 30 Days in Cold Chamber	After 45 Days in Cold Chamber	After 45 Days in Cold Chamber
Slow release—4 g	0.75 ± 0.12 a	1.53 ± 0.14 a	8.28 ± 0.21 a
Slow release—7 g	0.79 ± 0.14 a	1.53 ± 0.17 a	8.71 ± 0.29 a
Dual release—5 g	0.58 ± 0.12 a	1.08 ± 0.19 a	8.56 ± 0.13 a
Dual release—8 g	0.56 ± 0.16 a	1.30 ± 0.19 a	8.25 ± 0.11 a
**Ventilation area of perforated plastic liners (B)**			
0.3%	0.51 ± 0.11 b	0.99 ± 0.18 b	8.60 ± 0.20 a
0.9%	0.86 ± 0.10 a	1.81 ± 0.10 a	8.14 ± 0.15 a
1.0%	0.63 ± 0.16 ab	1.27 ± 0.16 b	8.61 ± 0.21 a
**Contrast of the additional treatment with the factorial**			
Additional treatment	0.91 ± 0.23 a	1.57 ± 0.25 a	8.73 ± 0.28 a
Factorial	0.67 ± 0.13 a	1.36 ± 0.18 a	8.45 ± 0.20 a
F (A)	1.09 ^ns^	2.93 ^ns^	1.57 ^ns^
F (B)	3.25 *	13.16 *	3.28 ^ns^
F (A × B)	1.68 ^ns^	1.95 ^ns^	1.77 ^ns^
F (additional treat. × factorial)	1.34 ^ns^	0.92 ^ns^	0.82 ^ns^
CV (%)	19.22	17.10	7.92

Means within columns followed by the same letters are not statistically different as determined by Tukey’s test (* *p* ≤ 0.05). ns: not significant. CV: coefficient of variation. Original data of mass loss were transformed by $\sqrt{x+0.5}$.
